# Supplementary material for: Stacking Model‐Based Classifiers for Dealing With Multiple Sets of Noisy Labels
Source: Biom J. 2025 Mar 12;67(2):e70042. doi: 10.1002/bimj.70042 (PMC11898607; doi:10.1002/bimj.70042)
Supplement: Supplementary file 1 — Supporting Information [file BIMJ-67-e70042-s001.zip › density-based-ensemble-model-main/competitors_real_data/netlab-master/netlab-master/help/glmhess.htm]

Netlab Reference Manual glmhess


# glmhess

## Purpose

Evaluate the Hessian matrix for a generalised linear model.

## Synopsis

```
h = glmhess(net, x, t)
[h, hdata] = glmhess(net, x, t)
h = glmhess(net, x, t, hdata)
```

## Description

`h = glmhess(net, x, t)` takes a GLM network data structure `net`,
a matrix `x` of input values, and a matrix `t` of target
values and returns the full Hessian matrix `h` corresponding to
the second derivatives of the negative log posterior distribution,
evaluated for the current weight and bias values as defined by
`net`. Note that the target data is not required in the calculation,
but is included to make the interface uniform with `nethess`. For
linear and logistic outputs, the computation is very simple and is
done (in effect) in one line in `glmtrain`.

`[h, hdata] = glmhess(net, x, t)` returns both the Hessian matrix
`h` and the contribution `hdata` arising from the data dependent
term in the Hessian.

`h = glmhess(net, x, t, hdata)` takes a network data structure
`net`, a matrix `x` of input values, and a matrix `t` of
target values, together with the contribution `hdata` arising from
the data dependent term in the Hessian, and returns the full Hessian
matrix `h` corresponding to the second derivatives of the negative
log posterior distribution. This version saves computation time if
`hdata` has already been evaluated for the current weight and bias
values.

## Example

The Hessian matrix is used by `glmtrain` to take a Newton step for
softmax outputs.

```
Hessian = glmhess(net, x, t);
deltaw = -gradient*pinv(Hessian);
```

## See Also

`glm`, `glmtrain`, `hesschek`, `nethess`

---

**Pages:**
Index


---

Copyright (c) Ian T Nabney (1996-9)
